# Serial magnetic resonance imaging changes of pseudotumor lesions in retinal vasculopathy with cerebral leukoencephalopathy and systemic manifestations: a case report

**DOI:** 10.1186/s12883-021-02250-4

**Published:** 2021-06-09

**Authors:** Yuying Yan, Shuai Jiang, Ruilin Wang, Xiang Wang, Peng Li, Bo Wu

**Affiliations:** 1 Department of Neurology, West China Hospital, Sichuan University, No. 37 Guo Xue Xiang, 610041 Chengdu, China; 2 Department of Ophthalmology, West China Hospital, Sichuan University, No. 37 Guo Xue Xiang, Chengdu, 610041 China; 3 Department of Neurosurgery, West China Hospital, Sichuan University, No. 37 Guo Xue Xiang, 610041 Chengdu, China

**Keywords:** Retinal vasculopathy with cerebral leukoencephalopathy and systemic manifestations, Hereditary cerebral small vessel disease, Three-prime repair exonuclease 1 gene, Tumefactive brain lesion, Case report

## Abstract

**Background:**

Retinal vasculopathy with cerebral leukoencephalopathy and systemic manifestations (RVCL-S) is an adult-onset rare monogenic microvasculopathy. Its typical neuroimaging features are punctate white matter lesions or pseudotumor alterations. RVCL-S is often under-recognized and misdiagnosed because of its rarity and similar imaging manifestations to multiple sclerosis or brain malignant mass.

**Case presentation:**

Here we report a case of a 36-year-old Chinese man who developed multiple tumefactive brain lesions spanning over two years leading to motor aphasia, cognitive decline, and limb weakness. He also presented with slight vision loss, and fundus fluorescein angiography indicated retinal vasculopathy. He underwent brain biopsies twice and showed no evidence of malignancy. Given the family history that his father died of a brain mass of unclear etiology, RVCL-S was suspected, and genetic analysis confirmed the diagnosis with a heterozygous insertion mutation in the three-prime repair exonuclease 1 gene. He was given courses of corticosteroids and cyclophosphamide but received little response.

**Conclusions:**

The present case is one of the few published reports of RVCL-S with two-year detailed imaging data. Serial magnetic resonance images showed the progression pattern of the lesions. Our experience emphasizes that a better understanding of RVCL-S and considering it as a differential diagnosis in patients with tumefactive brain lesions may help avoid unnecessary invasive examinations and make an earlier diagnosis.

## Background

Retinal vasculopathy with cerebral leukoencephalopathy and systemic manifestations (RVCL-S) is an exceedingly rare hereditary cerebral small vessel disease caused by mutations in the gene encoding the three-prime repair exonuclease 1 (TREX1) [1]. No more than 200 individuals have been identified worldwide so far [2]. RVCL-S patients exhibit a core constellation of neurological and visual symptoms. Brain magnetic resonance imaging (MRI) has shown the presence of punctate white matter lesions or tumor-like alterations in these patients [3]. The underlying mechanisms and disease progression patterns are ambiguous. Although therapeutic options for RVCL-S are limited, early diagnosis may prevent excessive invasive examinations.

Here we describe an RVCL-S patient who was initially misdiagnosed with tumefactive demyelinating lesions (TDLs). Two years’ serial neuroimages provided a detailed lesion evolution of the disease.

## Case presentation

A 36-year-old Chinese man presented with 26 months of progressive motor aphasia and cognitive decline. Fourteen months after the initial symptoms, he began complaining of mild right-sided weakness. He was admitted to our hospital for acute exacerbation of confusion and weakness of limbs lasting 3 days. Upon further questioning after admission, the patient indicated that he had been experiencing slight vision loss for 11 months, particularly the right one, which had not attracted any attention due to the ametropia of both eyes. Neurological examination revealed bradyphrenia, motor aphasia, normal pupillary response but decreased visual acuity of bilateral eyes (visual acuity could not be measured using the standard logarithmic visual acuity chart due to the poor consciousness state of the patient), right central facial palsy, dysarthria, and quadriplegia.

His medical history included papillary thyroid cancer for over 1 year without any special treatment and chronic hepatitis B virus infection and cirrhosis for 10 years under regular anti-virus therapy. The patient had no siblings. Family medical history revealed that his father had developed a brain mass of unclear etiology and died at the age of 34. While his mother did not suffer from any neurological disorder.

**Fig. 1:**
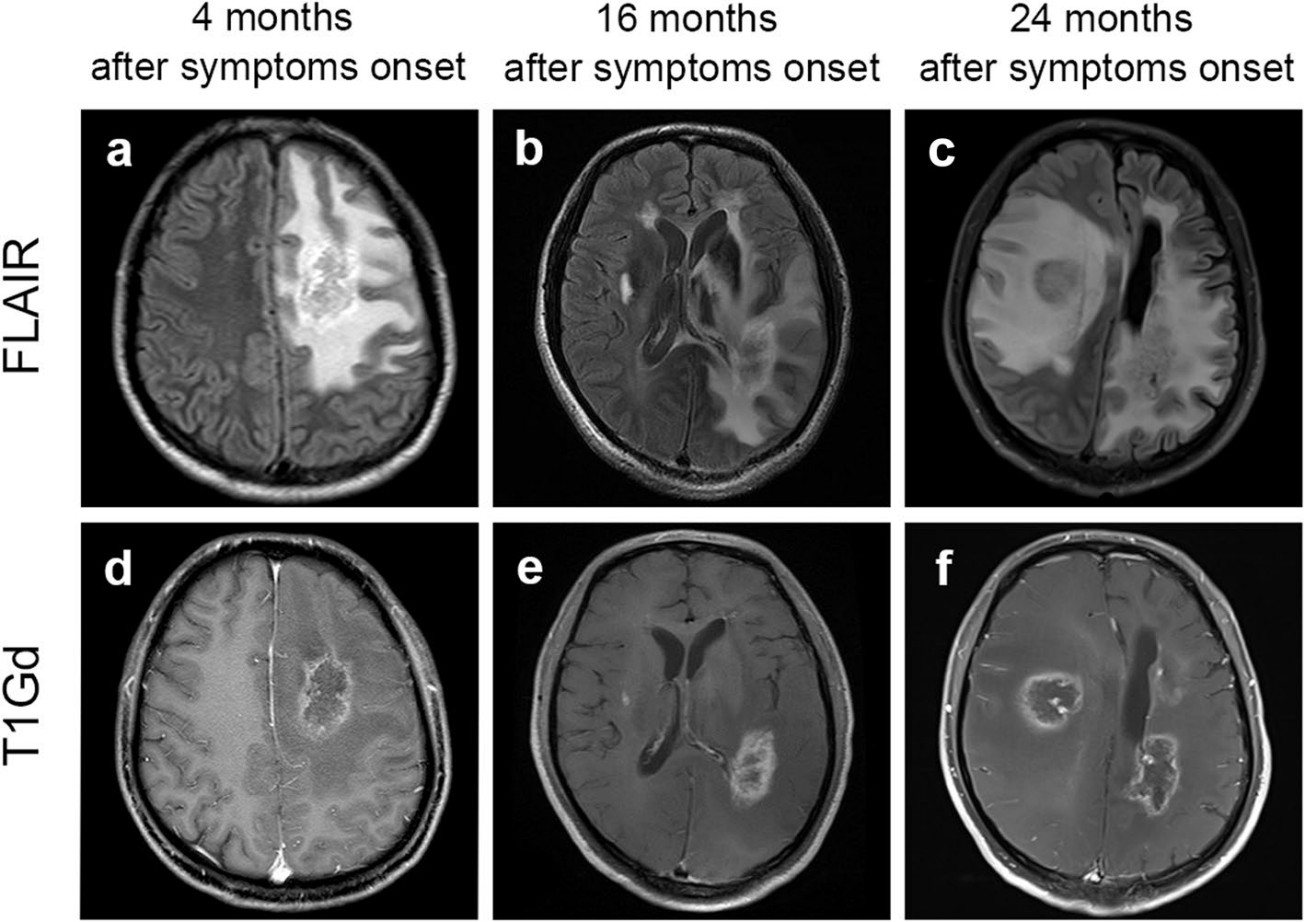
Serial brain magnetic resonance images (MRIs) of the patient during two years. Fluid attenuated inversion recovery (FLAIR) and T1-weighted gadolinium-enhanced (T1Gd) axial images were acquired at 4 (**a, d**), 16 (**b, e**), and 24 (**c, f**) months after symptoms onset. Images were taken 4 months after symptoms onset (**a, d**) and showed a closed-ring enhanced lesion with perilesional edema. Images acquired at month 16 (**b, e**) and 24 (**c, f**) revealed new lesions in the left temporal-occipital lobe and right temporal lobe. The right one progressed from a nodular enhancing lesion to a rim-enhancing lesion with edema

The first MRI and brain biopsy were conducted 22 months ago in another hospital. We observed a T2 hyperintense massive subcortical lesions with rim enhancement in the left frontal lobe (Fig. 1a and d) and a non-enhanced nodular lesion in the left cerebellum. Histopathology of the former lesion indicated focal necrosis and reactive gliosis, along with vessel wall hyalinization and inflammatory cell infiltration. The patient was tentatively diagnosed with TDLs 18 months ago. Three courses of high-dose intravenous methylprednisolone and two courses of intravenous cyclophosphamide had been given subsequently during the following 16 months. The patient received repeated brain MRIs every six months. Cerebral edema reduced while contrast enhancement still existed and a surgical trail was left in the frontal lesion. The new lesions appeared in the left temporal-occipital lobe and the right temporal lobe on serial images (Fig. 1b, c, e, and f) and gradually deteriorating clinically indicated these treatments failed to prevent relapse.

**Fig. 2:**
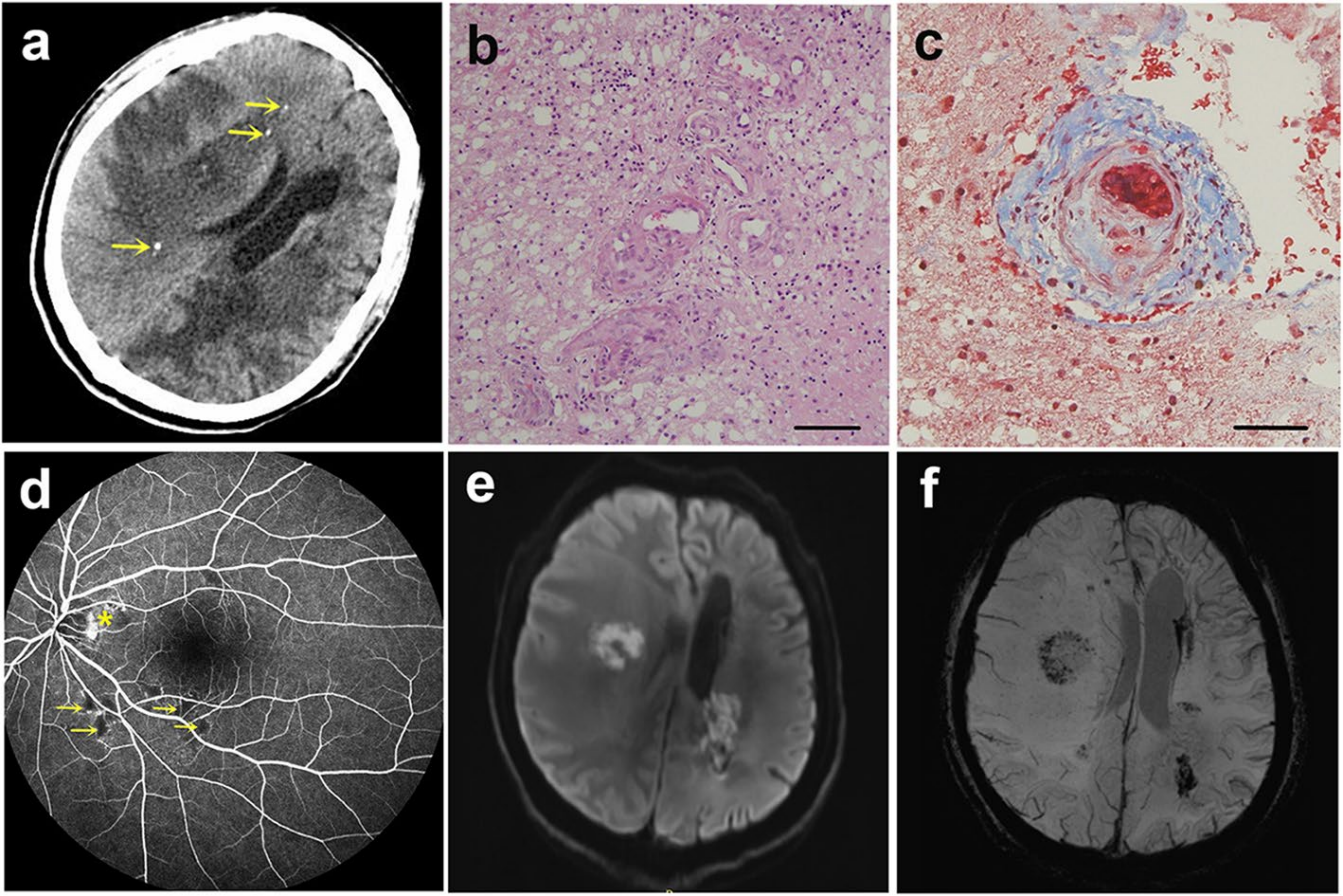
Some auxiliary examination results of the patients. Non-contrast computed tomography of the head showed predominant right temporal hypodensity with extensive edema and several calcifications (yellow arrows) (**a**). Hematoxylin- and eosin-stained histological section of the white matter of the brain (scale bar, 100 µm) revealed leukoaraiosis and neuropil edema, infiltrated by gitter cells, as well as vessel wall thickening with varying degrees of luminal narrowing. Some vessels were surrounded by lymphocytes (**b**). Masson stain of brain section (scale bar, 50 µm) showed small artery wall thickening with a proliferation of collagen fibers (blue) and formation of hyaline thrombi (**c**). Fluorescein angiography revealed extensive areas of capillary obliteration with non-perfusion (yellow arrow), and profuse leakage from the membrane on the disc (yellow asterisk) (**d**). Diffusion weighted image of the brain showed central diffusion restriction of the lesions in left temporal-occipital lobe and right temporal lobe (**e**). Susceptibility weighted axial images showed microbleeds in the lesions (**f**)

**Fig. 3:**
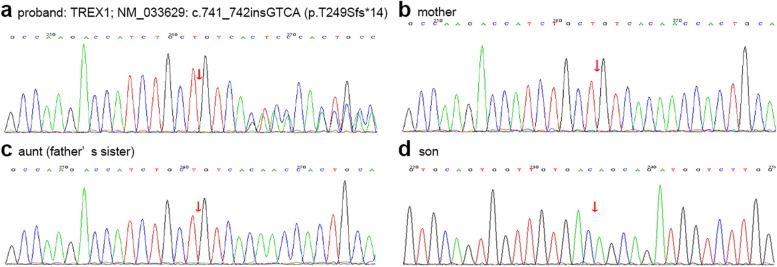
Sanger sequencing of TREX1 gene in the family. The proband carried a heterozygous mutation of TREX1 gene [TREX1; NM_033629: c.741_742insGTCA (p.T249Sfs*14)] (red arrow) **(a)**. The mother **(b)**, aunt **(c),** and son **(d)** of the proband lacked the mutation

Current laboratory examination revealed mild anemia, mildly elevated liver enzyme levels, and elevated thyroid autoantibodies. Cerebrospinal fluid (CSF) analysis showed normal pressure and only slightly elevated protein. No antibodies associated with ganglioside or autoimmune encephalitis (against NMDA-R, CASPR2-R, AMPA1-R, AMPA2-R, LGI1, GABA_B_-R, DPPX) or paraneoplastic neurologic syndromes (against CV2/CRMP5, PNMA2, Ri, Yo, Hu, Amphiphysin) were detected in CSF and serum. CSF oligoclonal band, serum AQP4-IgG, and MOG-IgG were not detected. Whole-body ^18^F-fluorodeoxyglucose-positron emission tomography/computed tomography showed no evidence of malignancy. Repeated non-contrast computed tomography (CT) of the head showed hypodense lesions and several punctate calcifications (Fig. 2a). A second brain biopsy of the lesion in the left temporal-occipital lobe was obtained but only revealed a similar result to the first time (Fig. 2b and c). We confirmed retinal vasculopathy using fundus photography and fluorescein angiography (Fig. 2d), leading us to suspect hereditary vasculopathy. Whole exome sequencing identified a heterozygous GTCA insertion (c.741_742insGTCA) in the TREX1 gene (NM_033629) resulting in a frameshift mutation (p.T249Sfs*14). Nucleotide change was confirmed by Sanger sequencing (Fig. 3a). Therefore, we diagnosed the patient with RVCL-S. Genetic testing of the mother, aunt (father’s sister), and son showed that all lacked the mutation (Fig. 3b, c, and d).

## Discussion and conclusions

RVCL-S is an adult-onset, autosomal dominant disease of small vessels caused by mutations that result in translocation of the normally perinuclear TREX1 DNA exonuclease into the cytoplasm and the nucleus [1, 4]. The dissemination of TREX1 may lead to failure of granzyme A apoptosis and bring detrimental effects [1]. RVCL-S typically presents as a core constellation of neurological and ophthalmological manifestations, while the liver, kidney, skin, and other organs can also be affected [3, 5].

All the typical lesion features of RVCL-S described in the prior research, including small punctate lesions with or without nodular enhancement and rim-enhancing pseudotumor lesions [3], could be observed on sequential neuroimages of the present case. The lesions were located in both supratentorial and infratentorial white matter areas. Periventricular lesions showed remarkably larger sizes than the cerebellar ones. For those lesions with aggressive appearance, central diffusion restriction (Fig. 2e), peripheral rim-enhancement and microbleeds (Fig. 2f), and massive perilesional edema could be found and persistent for several months or even longer.

Understanding the temporal-spatial dynamic evolution pattern of the lesion may give insight into mechanisms and treatment. Given the rarity of RVCL-S, studies on lesion progression are limited. One study based on serial MRI scans of six RVCL-S patients indicated the long duration of diffusion restriction and rim-enhancement of lesions [6]. Another recent study compared the MRI images between RVCL-S patients and healthy controls and concluded that white matter atrophy was a prominent feature in RVCL-S [2]. However, few studies built up the relation between different lesion categories. In the present case, the sequential MRI images demonstrated the progression from non-enhancing to enhancing lesions and nodular enhancing lesions to rim-enhancing lesions with edema. Contrarily, a previous report demonstrated the possibility of the shrunk of edema and the change of blood-brain barrier disruption from rim-enhancement to nodular appearance [6]. These transformations may indicate that these three features represent different stages of the disease and can not be separated.

Tumor-like lesions on brain MRI in RVCL-S can easily be misdiagnosed as TDLs, central nervous system (CNS) vasculitis, primary CNS lymphoma, or high-grade glioma. Most of these diseases can respond to corticosteroid treatment. In TDLs patients, postcorticosteroids resolution can be observed after 6–12 weeks, and the course of enhancement rarely persistent for more than 6 months. Brain neoplasms often obtained an initial response, but will subsequently progress. Moreover, tumefactive demyelinating lesions are usually surrounded with less substantial edema [7]. Our patient, who was initially misdiagnosed with TDLs based on radiologic and biopsy findings failed to respond to corticosteroids therapy and showed vascular retinopathy with a probable positive family medical history, leading us to suspect RVCL-S. Our case also suggests that focal white matter calcifications may help to diagnose RVCL-S. Those dystrophic calcifications located in the white matter area combined with abnormal vessel walls rather than directly in the vessel walls from the pathological view were easily recognized on CT scans [3, 8].

There is no specific therapeutic strategy for RVCL-S due to unclear and challenging mechanisms. The role of neuroinflammation and autoimmune played in the disease initiation and progression is still controversial. The phenomenon of inflammatory lymphocytic infiltrates in brain biopsies could be interpreted as a reaction to focal ischemia or the primary contribution to lesion pathogenesis [5]. In the other three disorders, Aicardi-Goutières syndrome, familial chilblain lupus, and systemic lupus erythematosus, due to TREX1 mutations, a type I interferon response is likely to be induced. Distinct from them, conventional immune phenotypes or serum interferon-associated inflammation markers are rarely detected in RVCL-S [4, 5]. One patient reported in a previous case identified an upregulation of type I interferon-dependent genes [9], which might support the theory of autoimmune activation in RVCL-S and become the treatment target. Despite the uncertainty of the pathogenesis of the disease, immunoregulation or immunosuppressive treatment is often given empirically. Corticosteroids are frequently used, but they can reduce vasogenic edema only temporarily and do not prevent relapses [10]. The same condition can be observed in the present case. The efficacy of antiproliferative or biological agents such as cyclophosphamide, azathioprine, and natalizumab is controversial. Tofacitinib and hydroxychloroquine may be effective because they reduce intracellular levels of interferon-α and -β [11]. Future studies should focus on these therapeutic agents.

The present case is one of the few published reports of RVCL-S with detailed imaging data of two years. Our experience highlights the need to consider RVCL-S as a differential diagnosis in patients with tumefactive brain lesions, which may help avoid unnecessary invasive examinations.

## Data Availability

All data related to this case report are documented within this manuscript.

## Abbreviations

RVCL-S: Retinal vasculopathy with cerebral leukoencephalopathy and systemic manifestations
TREX1: Three-prime repair exonuclease 1
MRI: Magnetic resonance imaging
TDLs: Tumefactive demyelinating lesions
CSF: Cerebrospinal fluid
CT: Computed tomography
CNS: Central nervous system
